# Disentangling within- and between-subject correlations in cognitive models: The essential role of hierarchical estimation

**DOI:** 10.3758/s13428-026-03111-z

**Published:** 2026-08-05

**Authors:** Michelle C. Donzallaz, Niek Stevenson, Andrew Heathcote, Dora Matzke

**Affiliations:** 1 Psychological Methods, Department of Psychology, University of Amsterdam, Amsterdam, The Netherlands; 2 School of Psychology, University of Newcastle, Callaghan, Australia

**Keywords:** Correlations, Bayesian hierarchical modeling, Individual differences, Cognitive models, Evidence-accumulation models

## Abstract

Cognitive models, such as evidence-accumulation models, are increasingly used in individual differences research in psychology and neuroscience. By computing correlations between cognitive model parameters across participants, researchers aim to understand how the psychological processes the parameters represent relate to one another and jointly determine performance. It is generally acknowledged that cognitive models can be challenging to estimate due to strong within-subject correlations among the parameters, which are embedded in the model’s likelihood function. What is less often recognized, however, is that within-subject correlations can also distort correlations computed between parameters estimated with non-hierarchical methods, so they no longer reflect true individual differences, potentially leading to misleading conclusions. Here we illustrate this pitfall of non-hierarchical estimation and show how appropriately parameterized, descriptively adequate hierarchical models can mitigate the problem by effectively separating within- and between-subject sources of variation. We then offer recommendations for identifying and guarding against the inferential biases resulting from the strong within-subject correlations inherent in many cognitive models.

Cognitive models have become increasingly popular for analyzing choice and response time (RT) data from experimental tasks (Farrell & Lewandowsky, 2018; Lee & Wagenmakers, 2014). These models are commonly used to explain and predict task performance through parameters that represent psychological processes involved in cognitive functions, such as decision making, attention, and memory (e.g., Erdfelder et al., 2009; Heathcote & Love, 2012; White, Ratcliff, & Starns, 2011). Beyond providing formal theoretical accounts of the processes underlying performance, many cognitive models also serve as measurement tools for assessing individual differences through correlations between their parameters (Donzallaz et al., 2026; Forstmann, Ratcliff, & Wagenmakers, 2016; van der Maas, Molenaar, Maris, Kievit, & Borsboom, 2011).

Consider, for instance, the diffusion decision model (DDM; Donkin & Brown, 2018; Ratcliff & McKoon, 2008; Ratcliff & Smith, 2004; Wagenmakers, 2009), a prominent cognitive model used to account for choices and associated RTs in rapid two-choice decision-making tasks. The model conceptualizes decision-making as a gradual, noisy accumulation of evidence toward one of two response boundaries. Once a boundary is reached, the corresponding response is initiated. Figure 1 illustrates the DDM and its four main parameters: 1) drift rate *v*, which quantifies the mean rate of evidence accumulation, reflecting the quality and speed of information processing; 2) boundary separation *a*, representing the distance between the two response boundaries, reflecting response caution; 3) *z*, which quantifies the start point of evidence accumulation, reflecting response bias; and 4) non-decision time *t*0, capturing the time required for processes outside the decision itself, such as stimulus encoding and motor execution.

**Fig. 1:**
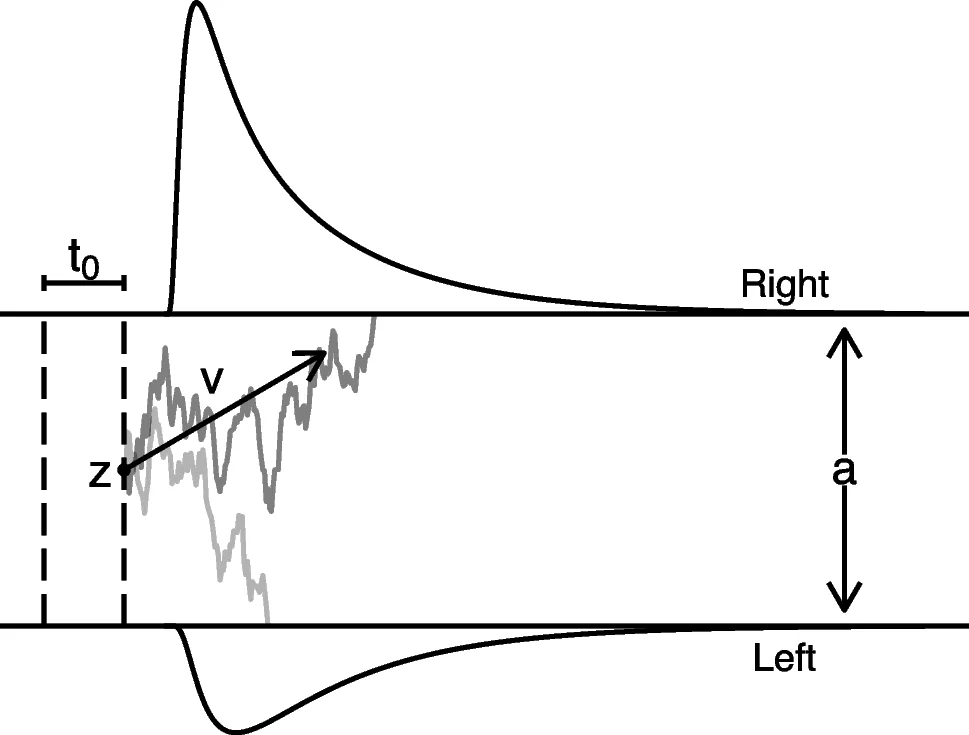
The diffusion decision model (DDM) for choosing between two options (e.g., “left” vs. “right” in a random dot motion task) and its four main parameters. The example illustrates the model’s behavior for a stimulus in which “right” is the correct response. Following stimulus onset, evidence accumulation begins at the starting point *z* and continues at a mean drift rate *v*, reflecting the quality and speed of information processing. This accumulation continues until one of two decision boundaries is reached: the upper, correct boundary or the lower, incorrect boundary, at which point a response is triggered. Boundary separation *a* is the distance between the two boundaries, reflecting response caution. Wider boundaries imply more cautious decision-making. Non-decision time *t*0 reflects the duration of processes outside the decision itself, such as stimulus encoding and motor execution. The figure includes two exemplary evidence trajectories, one terminating at the correct, upper boundary and one at the incorrect, lower boundary. This process, repeated across many trials, gives rise to the response time distributions for correct and incorrect responses, depicted above the upper boundary and below the lower boundary, respectively

Now, suppose a researcher applies the DDM to a random-dot motion task in which participants are presented with a cloud of dots on each trial. A subset of the dots moves coherently in one direction and the rest moves randomly. The task is to indicate the direction of the coherently moving dots (i.e., left or right). The experiment includes a within-subject difficulty manipulation: On *Easy* trials, the proportion of dots moving in the same direction is high, making it fast and easy to identify the motion direction. On *Hard* trials, the proportion is lower, resulting in slower and less accurate responses.

After fitting the DDM to the choice RT data of each participant separately, the researcher finds higher response caution and longer non-decision time in the *Hard* condition than in the *Easy* condition. These effects are captured by positive difference scores: $a_{d} = a_{hard}-a_{easy}$ and $t0_d = t0_{hard}-t0_{easy}$. To assess the degree to which individual differences in the two difference scores are related, the researcher computes and tests the correlation between participants’ $a_{d}$ and $t0_d$ estimates. Although this two-step approach – first estimating parameters using a model and then using these parameters for statistical inference – is common in the literature, it has been shown to yield overconfident and at times misleading conclusions (Boehm, Marsman, Matzke, & Wagenmakers, 2018; Ly et al., 2017), including in other disciplines (e.g., econometrics; Pagan, 1984).

For cognitive models such as the DDM, the limitations of the two-step approach are further compounded by the fact that the model parameters may be correlated not only at the between-subject level but also within participants (Ratcliff & Tuerlinckx, 2002). This also applies to differences between parameters, as used in our running example. Between-subject correlations reflect the degree to which individual differences in the two sets of model parameters are related to each other. For example, individuals who tend to respond more cautiously, as reflected in higher boundary separation *a*, may also take more time to encode the stimulus and initiate a response, as reflected in higher non-decision time *t*0. Within-subject correlations, in contrast, reflect parameter dependencies that arise from a model’s functional structure. During parameter estimation, these mathematical dependencies in the likelihood of the model cause parameters to trade off, leading to correlated deviations from their true values (Schmiedek, Oberauer, Wilhelm, Suss, & Wittmann, 2007). In extreme cases, a perfect within-subject correlation renders parameters non-identifiable.

Cognitive models in general, and evidence-accumulation models such as the DDM in particular, are known to have pronounced within-subject parameter trade-offs, a feature sometimes referred to as *sloppiness* (Gutenkunst et al., 2007). For example, in the DDM, there is a pronounced negative within-subject correlation between *t*0 and *a*, and a positive within-subject correlation between *v* and *a*, especially for low error rates (Lüken, Heathcote, Haaf, & Matzke, 2025). Such structural dependencies in the model are a core part of the theory instantiated by a cognitive model, but they are not the focus of inference in individual differences studies. Although sloppiness is generally acknowledged in the cognitive modeling community (e.g., Krefeld-Schwalb, Pachur, & Scheibehenne, 2022; Scheibehenne & Pachur, 2015), it is less widely recognized – with a few notable exceptions (e.g., Ratcliff & Tuerlinckx, 2002; Schmiedek et al., 2007) – that observed correlations between parameter estimates obtained via two-step approaches do not necessarily reflect true between-subject correlations. Instead, in sloppy models, they can be severely contaminated by within-subject parameter trade-offs.

**Fig. 2:**
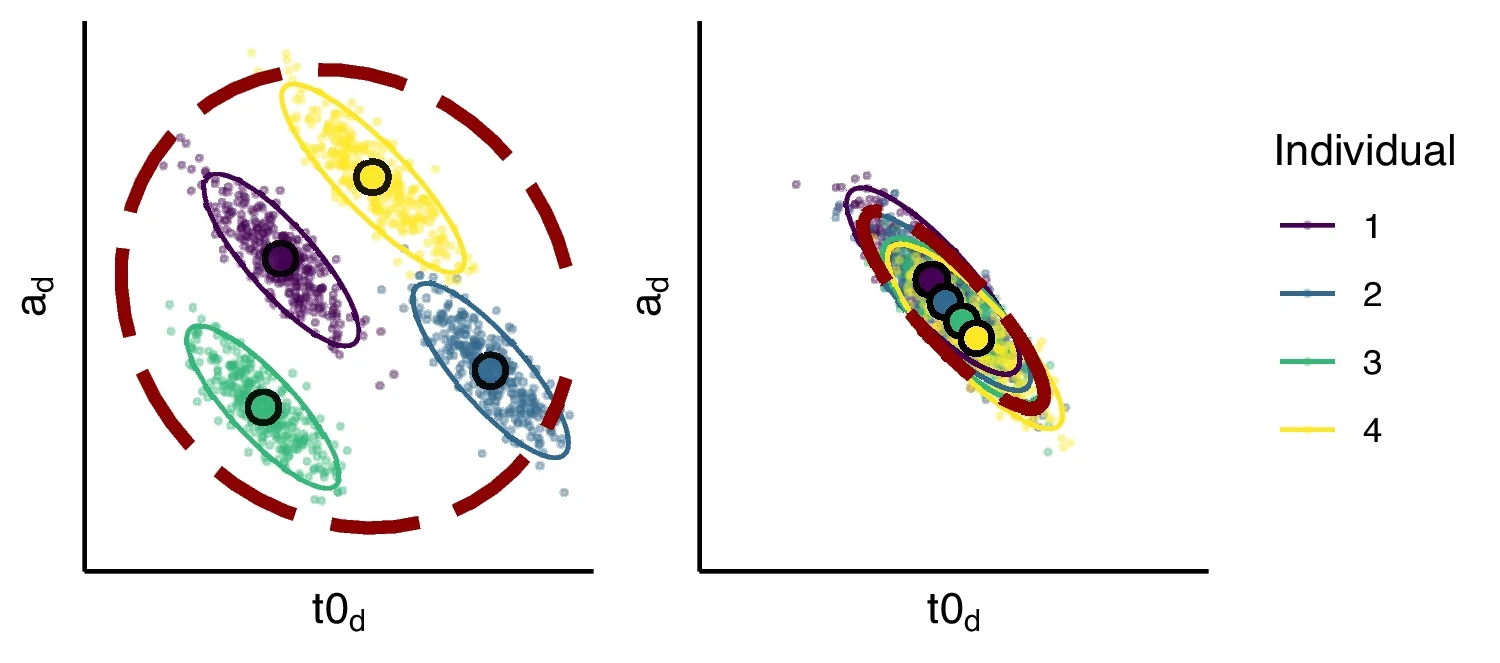
An illustration of between- and within-subject correlations in the presence (*left*) and absence (*right*) of true between-subject variation, using the DDM difference scores $t0_{d}$ and $a_{d}$ as an example. The *ellipses* represent within-subject variability (i.e., trade-off) corresponding to the parameter estimates of four artificial participants. The *central dots* represent the means. In the left panel, there is between-subject variability in both $t0_{d}$ and $a_{d}$. Although the parameters show strong trade-offs within participants, their between-subject correlation is negligible (see the *dark red ellipse*). In the right panel, there is no between-subject variability in either parameter, causing individual ellipses to heavily overlap. The small differences between the ellipses are purely due to correlated measurement error, which introduces variability in the estimates even when the data-generating parameters are the same for all participants. This, in turn, causes the observed correlation between parameter estimates to mirror the within-subject correlation

The fact that between- and within-subject correlations operate on different levels and may even differ in direction poses challenges for accurate interpretation. This is illustrated by a recent study, which provides an example of how within-subject correlations can lead to misinterpretations of observed correlations between parameter estimates (Grange & Schuch, 2023). Using the two-step approach, the authors observed strong negative correlations (-.70 and higher) between difference scores $a_{d}$ and $t0_d$ derived from the DDM fit separately to two conditions of a within-subject design – even though the true between-subject correlation was zero. The authors demonstrated that their results were robust across various estimation methods and software packages, and were present both in real and simulated data. This led them to suggest that “[...] the difference scores in these two model parameters should not be used in individual-differences research, because they do not necessarily reflect true inter-individual differences at the population level” (p. 3351). As we show here, making valid inferences about individual differences in cognitive model parameters requires accounting for the interaction between within-subject and between-subject correlations.

The goal of the present paper is to highlight how and when within-subject correlations can masquerade as between-subject correlations, and to provide a solution to this statistical artifact. We use Grange and Schuch (2023) as a case study to show when the commonly used two-step approach can lead to spurious conclusions and how hierarchical modeling effectively addresses these problematic cases. We aim to show that – contrary to Grange and Schuch ’s conclusions – correlations among evidence-accumulation model parameters, as well as their differences, can be safely used in individual differences research, and to outline how to do so reliably.

## Between vs. within-subject correlations

The interplay of between- and within-subject variability in correlations has long been recognized in time series analyses – where within-subject variability stems from fluctuations over multiple measurement occasions and between-subject variability from stable individual traits (Fisher, Medaglia, & Jeronimus, 2018; Molenaar, 2004). A clear illustration is provided by Hamaker (2012) using the correlation between typing speed and typos. At the between-subject level, the correlation is negative: people who type faster make fewer mistakes. However, within individuals, the pattern is reversed. The faster one types, the more mistakes one makes.

The correlations computed across participants at a given time point (i.e., cross-sectional correlation) can be expressed as a weighted sum of the between- and within-subject correlation (Dansereau, Alutto, & Yammarino, 1984; Hamaker, 2024; Hsu, Poldrack, Ram, & Wagner, 2022; Robinson, 1950). The weights are determined by the intraclass correlations (ICC) of the two variables, that is the respective proportions of total variance that can be attributed to stable individual differences. Hamaker (2024) showed, using simulations, that when there is no between-subject variability in either variable *X* or *Y*, the cross-sectional correlation between the two is the same as their within-subject correlation. When there is between-subject variability in both *X* and *Y* but no between-subject correlation, the cross-sectional correlation lies between the between-subject correlation (i.e., zero) and the within-subject correlation.

Similar principles apply to correlations involving parameters of sloppy cognitive models. Between-subject correlations represent true individual differences in the model parameters, while within-subject correlations reflect how the model parameters co-vary (or trade off) within a participant. As we show later, analogous to cross-sectional correlations, correlations computed between parameter estimates from two-step approaches can also be expressed as a weighted sum of within- and between-subject correlations. This implies that the computed correlation may not purely reflect individual differences – or, in some cases, may not reflect individual differences at all. Figure 2 provides an intuition of this principle: When the true between-subject variability in model parameters is zero, the observed correlation between parameter estimates is the same as the within-subject correlation.

In cognitive models, within-subject correlations can be assessed through various methods, depending on the estimation approach used. If the first and second derivatives of a model’s likelihood function are available or can be approximated, within-subject correlations can be computed directly (e.g., Gutenkunst et al., 2007). Alternatively, they can be quantified using simulations: datasets are generated based on one fixed set of parameters and correlations calculated between the estimated parameters across the simulated datasets (e.g., Ratcliff & Tuerlinckx, 2002). Here, we focus on Bayesian estimation, where within-subject correlations are directly reflected in the joint posterior distribution of the parameters estimated using Markov chain Monte Carlo (MCMC) methods (Gamerman & Lopes, 2006; Lee & Wagenmakers, 2014).

### Safeguarding inference in cognitive models

To avoid spurious conclusions in DDM analyses, Ratcliff et al. (2011) suggested the heuristic of interpreting correlations between parameter estimates resulting from two-step methods only when observed individual variability is at least 2.5–5 times greater than within-subject variability. Using Monte Carlo simulations, Ratcliff and Tuerlinckx (2002) estimated this ratio by generating a large number of datasets from several fixed parameter sets in a particular design and fit the DDM to each one. Because within each parameter set, all datasets were based on the same underlying data-generating parameter values, variation in the estimates reflects only within-subject variability. This within-subject variability can then be compared to the observed individual variability in parameter estimates. If they are comparable in size, observed individual differences in parameter estimates (largely) represent noise and should not be used for inference. Because the extent of within-subject variability for a fixed parameter set decreases as a function of the square root of the number of test trials per condition per participant, Monte Carlo estimates of within-subject variability can be scaled against different trial numbers (Ratcliff et al., 2011). However, changes in experimental design or parameter set require new simulations. In practice, this makes it difficult to determine whether the extent of observed individual variability in empirical parameter estimates is sufficient to outweigh the within-subject variability (see also Haslbeck & Epskamp, 2023).

A more general approach, applicable not only across designs and parameter sets but also across different models, is to disentangle the two sources of (co-)variance using multivariate statistical methods, such as structural equation models (SEM). Specifically, Schmiedek et al. (2007) suggested using SEMs to extract latent factors of cognitive model parameters to capture their true between-subject correlation at the latent level, without distortion from within-subject correlations. First, cognitive model parameters are estimated separately for each participant across a set of tasks. The resulting parameter estimates then serve as indicators in the SEM and their loadings onto corresponding latent parameter factors are estimated. Crucially, both the latent factors as well as the error terms within the tasks are allowed to correlate. Because the within-task correlations are a combination of task-specific between-subject correlations and within-participant parameter trade-offs, the correlations among the latent parameter factors represent pure between-subject correlations among the cognitive model parameters, effectively disentangled from within-subject dependencies. However, the SEM approach requires data from multiple tasks and large sample sizes, which can be difficult to achieve in practice. It also assumes that the cognitive model parameters across tasks can be captured by the same latent factors, which may not always be the case. Finally, since it is a two-step procedure, this approach ignores uncertainty in the cognitive model parameter estimates, leading to overly confident inference (Stevenson, Heathcote, Forstmann, & Matzke, 2024).^1^

Here we outline an alternative solution that overcomes these limitations and efficiently separates within- and between-subject (co-)variance in cognitive models that suffer from strong parameter trade-offs: hierarchical modeling of correlations. Hierarchical (or multilevel) modeling (Gelman & Hill, 2007) assumes that participant-specific cognitive model parameters follow a multivariate (typically normal) distribution, and enables inference at two levels: the individual and the population. It provides cognitive model parameter estimates for each participant while simultaneously estimating between-subject variability and correlations among these parameters, encapsulated in the multivariate population-level distribution. Through this simultaneous estimation, and by explicitly accounting for between-subject (co-)variance, hierarchical modeling can effectively disentangle within- and between-subject correlations. Moreover, hierarchical modeling properly propagates estimation uncertainty from the participant to the population level, resulting in well-calibrated inference that does not inflate confidence in the results.

## Case study

In an extensive series of simulation studies and re-analyses of archival data, Grange and Schuch (2023) reported the pervasive emergence of spurious correlations – arising in the absence of true between-subject correlations – between difference scores computed from parameter estimates in evidence-accumulation models. Their investigation was motivated by observing a strong negative correlation between two estimates in their own datasets, specifically a correlation of around -.70 between the difference scores $t0_d$ and $a_d$. The difference scores were derived from a simple DDM (Stone, 1960) with parameters *a*, *t*0, and *v* in a within-subject design (*Hard* vs. *Easy* manipulation) using the two-step approach.

In Simulation 1, Grange and Schuch (2023) examined whether the spurious correlation emerges when no differences between the *Hard* vs. *Easy* experimental conditions are present in *a*, *t*0, and *v*. In Simulation 2, they investigated the case where a condition effect existed in one of the parameters. Simulation 3 replicated Simulation 1 using different software; Simulation 4 replicated Simulation 1 with the linear ballistic accumulator model (Brown & Heathcote, 2008), another popular evidence-accumulation model. Lastly, Simulation 5 examined whether a true correlation between difference scores could be recovered, if present. In additional analyses, the authors also explored the effects of high choice accuracy rates in the data, Bayesian hierarchical modeling, and latent change-score modeling. In a nutshell, across their simulations, Grange and Schuch found that the spurious correlation between $t0_d$ and $a_d$ emerged whenever no condition difference was simulated or where only the drift rate parameter (*v*) differed between simulated experimental conditions. They also reported similar results for two of their own studies (Schuch & Grange, 2019) and reanalyzed three experiments from the many-analysts project by Dutilh et al. (2019). In the latter, they found spurious correlations in two out of three datasets. Grange and Schuch (2023) suggested that spurious correlations between difference scores in *t*0 and *a* may be a general property of evidence-accumulation models. While they raised the possibility that parameter trade-offs could play a role, between- and within-subject correlations were not explicitly distinguished, and the role of their complex interplay in shaping the observed correlation between parameter difference scores under the two-step approach was not addressed.

Here, we replicate and extend Simulation 1 (and partly Simulation 2) as well as the reanalysis of Experiment 1 of Dutilh et al. (2019) to demonstrate that spurious correlations can be avoided with hierarchical modeling, and to clarify what this reveals about the fundamental differences between the within- and between-subject correlations. To foreshadow, we show that the simulation results reported in Grange and Schuch (2023) can be explained by an assumption of zero between-subject variation in $a_d$ and $t0_d$, in which case correlations computed between the difference scores fully reflect within-subject trade-offs between the parameters. In addition, in an extended reanalysis of Dutilh et al. (2019), we demonstrate that the spurious correlations disappear when using a descriptively adequate hierarchical model that jointly estimates participant-level cognitive model parameters and their covariance at the population level.

### Spurious observed correlations in the two-step approach

Grange and Schuch (2023)’s Simulation 1 and 2 used the procedure below, which we also adopted for our analyses, with a few notable differences.

#### Simulation and estimation procedure

 

1. **Parameter generation:** In Simulation 1, Grange and Schuch (2023) sampled 1000 individual parameter sets for a simple DDM (i.e., *a*, *t*0, and *v*)^2^ from uniform distributions in an *Easy* and a *Hard* condition. Importantly, within participants, the exact same parameters were used for the two conditions (e.g., $t0_{hard} = t0_{easy}$), so all participants had true difference scores of zero, with an intended between-subject correlation of zero and with no variability across participants: $\sigma _{Ba_{d}} = \sigma _{Bt0_{d}} = \sigma _{Bv_{d}}=0$). Note that the Pearson correlation coefficient *r* assumes finite and positive standard deviations: $r=\frac{cov(X, Y)}{\sigma _{X} \sigma _{Y}}$. With no individual variation in *X* or *Y*, the denominator equals zero, which means that in Simulation 1, the true between-subject correlations between the difference scores are mathematically undefined, and not zero as intended. Nevertheless, it is still possible to compute correlations between *estimates* of the difference scores because sampling variance and the associated estimation uncertainty will ensure that the variability of the estimated difference scores is larger than 0, even if the true data-generating $\sigma _{Ba_{d}}$ and $\sigma _{Bt0_{d}}$ are zero. In Simulation 2, condition differences between the DDM parameters were introduced. The authors systematically let one of the three parameters change within participants, varying the size of the condition difference at three levels, by simulating parameter sets from a univariate normal distribution for each condition. The two other parameters were still sampled from uniform distributions and used for both conditions, so they were not allowed to vary within participants, as in Simulation 1. This again leads to mathematically undefined correlations since in all combinations, one of the difference score variabilities $\sigma _{Ba_{d}}, \sigma _{Bt0_{d}}$, or $\sigma _{Bv_{d}}$ is always zero. To simulate data with the intended (i.e., mathematically defined) between-subject correlations of zero between the difference scores, we generated parameters for 1000 participants from a multivariate normal distribution. For the individual DDM parameters, we used the following parameterization: $v_{hard} = v_{easy} + v_{d}$, $a_{hard} = a_{easy} + a_{d}$, and $t0_{hard} = t0_{easy} + t0_{d}$^3^. All sampled parameters (*Easy* intercepts and difference scores) were generated from a multivariate normal distribution with means $\boldsymbol{\mu }$ and a diagonal variance-covariance matrix diag($\boldsymbol{\sigma ^2}$). This parameterization allowed us to explicitly simulate between-subject variability in the difference explicitly scores $a_d$, $t0_d$, and $v_d$ as well as setting the covariance terms to zero. We generated parameter sets with and without condition differences, as well as 11 different levels of between-subject variability in difference scores ranging from zero – as in Grange and Schuch ’s (2023) Simulation 1 – to $\sigma _{Ba_{d}} = 0.2, \sigma _{Bt0_{d}} = 0.1$, and $\sigma ^2_{Bv_{d}}=1$ (see Table 1)^4^. The extents of individual differences in $a_{d}$, $v_d$, and $t0_{d}$ are not well established in the literature. We therefore focused on choosing realistic values that resulted in individual parameters, both for the *Easy* and *Hard* conditions, that lie in the ranges reported in Tran, van Maanen, Heathcote, and Matzke (2021)^5^.

**Table 1:** Population means and standard deviations (SD) individual parameter sets with and without population-level group differences (indicated by “|”). The population means ($\mu _{v_{easy}}, \mu _{v_{d}},\mu _{a_{easy}},\mu _{a_{d}},\mu _{t0_{easy}}, \mu _{t0_{d}}$) were taken from Grange and Schuch (2023)’s Simulation 2 (we used the biggest condition difference; see their Table 6) since Simulation 1 generated data without condition differences. We used slightly smaller population standard deviations ($\sigma _{Bv_{easy}}, \sigma _{Bv_{d}},\sigma _{Ba_{easy}},\sigma _{Ba_{d}},\sigma _{Bt0_{easy}}, \sigma _{Bt0_{d}}$) to avoid generating undefined parameters on the natural scale (i.e., negative non-decision time $t_0$ or boundary separation *a*). The standard deviation of the difference scores varied across 11 equally spaced values between 0 and the indicated maximum, resulting in a total of 22 parameter settings (11 with and 11 without population-level condition differences)

Parameter	Population means (μ)	Population SD ($\sigma _B$)
$v_{easy}$	2.8	0.8
$v_{d}$	0 | -0.8	[0, 1]
$a_{easy}$	1.25	0.3
$a_{d}$	0 | 0.32	[0, 0.2]
$t0_{easy}$	0.35	0.05
$t0_{d}$	0 | 0.08	[0, 0.1]
  **Fig. 3:**
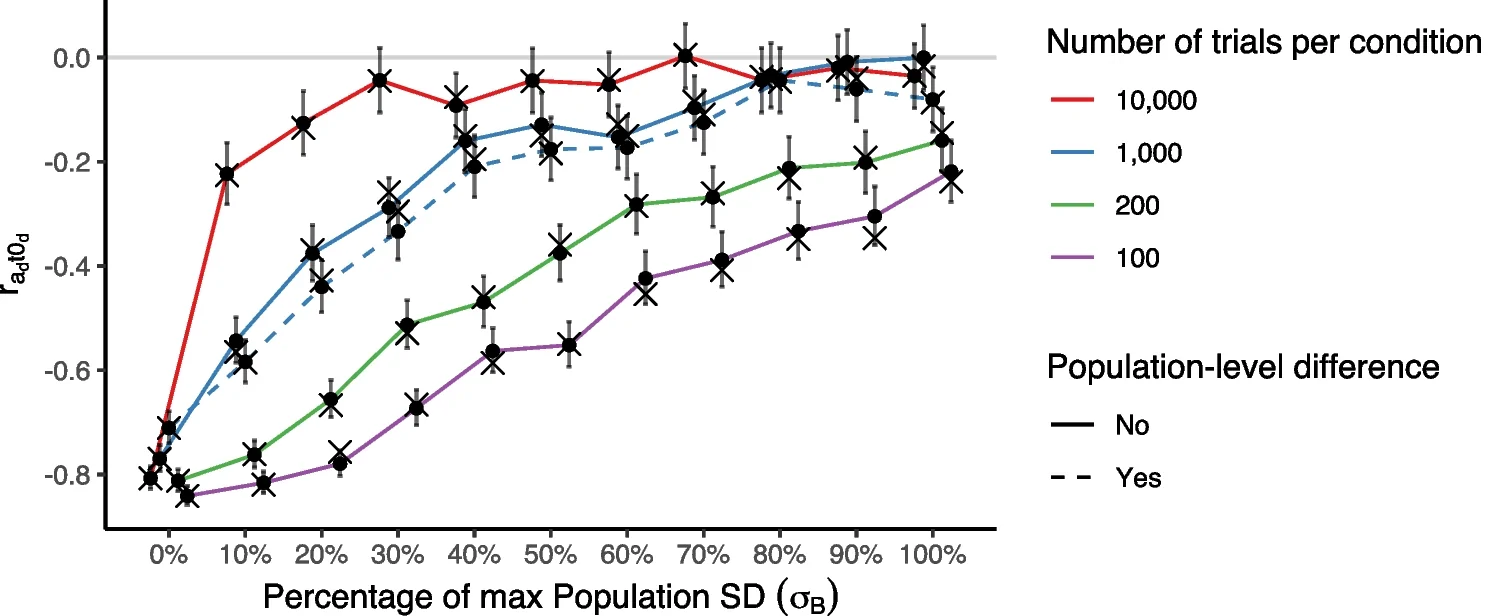
Observed correlations between estimates of $t0_{d}$ and $a_{d}$, computed using the two-step approach, and corresponding 95% confidence intervals as a function of the extent of between-subject variability (i.e., $\sigma _{Ba_{d}}$ and $\sigma _{Bt0_{d}}$), the number of trials per condition, and the presence or absence of a population-level mean difference between the *Easy* and the *Hard* conditions. Each *dot* represents one simulated dataset. At 0%, the data were simulated with no between-subject variability (i.e., $\sigma _{Ba_{d}}=\sigma _{Bt0_{d}}=0$), replicating the correlation reported by Grange and Schuch (2023). At 100%, they were simulated with the upper bound of the population standard deviation of the difference scores reported in Table 1. The *black crosses* are the correlations $r_{a_{d}t0_{d}}$ computed as a weighted sum of the true between-subject correlation (which is zero) and the within-subject correlation as observed at 0% between-subject variability in the difference scores
2. **Data generation:** Using the 1000 individual parameter sets, Grange and Schuch (2023) generated RT and choice data for the *Easy* and *Hard* conditions, with 1000 trials per condition per participant using the *construct-samples* routine of the software *fast-dm-30* (Voss, Voss, & Lerche, 2015). We extended the simulation to four levels of trial numbers per condition (100, 200, 1,000, and 10,000) and generated data using the R package *rtdists* (Singmann, Brown, Gretton, & Heathcote, 2022). For the generated data with 1000 trials, we additionally generated datasets with a condition difference as in Grange and Schuch (2023)’ Simulation 2. The error rates ranged from 5.2 to 6.8% across the datasets. Due to the different simulation setup, they are lower than in the data simulated by Grange and Schuch , where it was 15.4%. However, our error rates were still within the recommended range for the DDM (Lüken et al., 2025).
3. **Parameter estimation:** Grange and Schuch (2023) estimated DDM parameters separately for each condition and participant using both the *fast-dm-30* software (Voss et al., 2015) and EZ-diffusion estimation (Wagenmakers, van der Maas, & Grasman, 2007). We used EZ estimation for our simulations using Grange and Schuch (2023)’s R code (R Core Team, 2025)^6^. This approach estimates *a*, *v*, and *t*0 for an unbiased (z=a/2) simple DDM (Stone, 1960) based on a method-of-moments solution using the observed proportion of correct responses as well as the mean and variance of RT in a single condition.
4. **Difference score computation and correlational analysis:** Lastly, Grange and Schuch (2023) used parameter estimates for each participant to calculate difference scores between the *Easy* and *Hard* conditions, which they then correlated with each other using Pearson’s product-moment coefficient. In this step, they observed a correlation between estimates of $a_{d}$ and $t0_{d}$ of r= –.743 in Simulation 1, which they flagged as spurious given the absence of between-subject correlation in data-generating values. We followed their approach and calculated correlations between difference scores based on our EZ estimates.

#### Results

As shown in Fig. 3, we replicated the strong spurious correlation between estimates of $a_{d}$ and $t0_{d}$ in the absence of individual differences in $a_{d}$ and $t0_{d}$ (i.e., $\sigma _{Ba_{d}}=0$ and $\sigma _{Bt0_{d}}=0$). Specifically, with 1000 trials per condition, the observed correlation between the estimated difference scores is r=-.71, 95% confidence interval (CI) [ -.74, -.68], in the dataset with a population-level mean difference between *Easy* and *Hard* conditions, and r=-.76, 95% CI [ -.79, -.74], in the dataset without. The observed correlation decreases as a function of the extent of between-subject variability in the difference scores. That is, as $\sigma _{Ba_{d}}$ and $\sigma _{Bt0_{d}}$ increase, the correlation approaches its true value at zero, regardless of the presence or absence of a population-level mean difference between conditions. Figure 3 also shows that the number of trials per condition determines the rate at which the observed correlation approaches zero with increasing individual differences. The decrease is the steepest for 10,000 trials per condition and flattest for 100 trials per condition.

The observed correlation $r_{a_{d}t0_{d}}$ between $a_{d}$ and $t0_{d}$ can be expressed as a weighted sum of the within-subject correlation $\rho _W$ and the between-subject correlation $\rho _B$. Specifically, the weights are determined by the proportion of total variance $\sigma ^2_{T}$ that can be attributed to between-subject variance $\sigma ^2_{B}$ (see Hamaker, 2024, for the analytical derivation):

1 $$\begin{aligned} r_{a_{d}t0_{d}}=\eta _{\sigma _{Ba_{d}}}\eta _{\sigma _{Bt0_{d}}}\rho _{B}+\eta _{\sigma _{Wa_{d}}}\eta _{\sigma _{Wt0_{d}}}\rho _{W}, \end{aligned}$$

where $\eta _{Ba_{d}}=\sigma _{Ba_{d}}/{\sigma _{Ta_{d}}}$, $\eta _{Bt0_{d}}={\sigma _{Bt0_{d}}}/{\sigma _{Tt0_{d}}}$, and $\eta _{Wa_{d}}={\sigma _{Wa_{d}}}/{\sigma _{Ta_{d}}}$, $\eta _{Wt0_{d}}={\sigma _{Wt0_{d}}}/{\sigma _{Tt0_{d}}}$ are the weights of the between-subject correlation $\rho _{B}$ and of the within-subject correlation $\rho _W$, respectively. Note that the squared within-subject weights are the complement of the between-subject weights relative to the total variance:

2 $$\begin{aligned} \eta ^2_{Wt0_{d}} = 1-\frac{\sigma ^2_{Bt0_{d}}}{\sigma ^2_{Tt0_{d}}} \end{aligned}$$

3 $$\begin{aligned} \eta ^2_{Wa_{d}} = 1-\frac{\sigma ^2_{Ba_{d}}}{\sigma ^2_{Ta_{d}}}. \end{aligned}$$

Figure 3 shows the values calculated using this expression as black crosses, accurately tracking the simulation results. We computed $\eta _{Ba_{d}}$ and $\eta _{Bt0_{d}}$ as the ratio between the known true difference score variance and the variance of the difference scores as computed using the EZ estimates (i.e., the total variance). $\eta _{Wa_{d}}$ and $\eta _{Wt0_{d}}$ were calculated using Eqs. 2 and 3. The true between-subject correlation, $\rho _B$, is known to be zero. Finally, we computed $\rho _W$ by correlating the EZ difference scores obtained from datasets generated with $\sigma _{Ba_{d}}=\sigma _{Bt0_{d}}=0$; in the absence of between-subject variability, the within-subject correlation equals the observed correlation $r_{a_{d}t0_{d}}$ (Hamaker, 2024; Haslbeck & Epskamp, 2023).

#### Discussion

Our extended replication of Grange and Schuch (2023)’s Simulation 1 (and partly Simulation 2) using the EZ diffusion model showed that in the absence of between-subject variability in the difference scores ($\sigma _{Ba_{d}}=0,\sigma _{Bto_{d}}=0$), there is indeed a strong, negative spurious correlation between estimates of $a_{d}$ and $t0_{d}$. However, when we gradually increased the level of individual differences in both difference scores, the observed correlation gradually decreased and approached its true value (i.e., zero). These results are independent of whether or not there is a population-level mean difference between the two conditions.

We also showed analytically and through simulations, that the observed correlation is a weighted sum of the between- and within-subject correlations. The degree to which the within-subject correlation dominates the observed correlation depends on the number of trials: with more trials, the observed correlation will increasingly reflect the true between-subject correlation. Importantly, although the influence of within-participant variability can be reduced by increasing the number of trials, and thus increasing estimation precision, correlations computed between parameters of sloppy models will inevitably reflect a mixture of between- and within-subject correlations. The only exception is when the within-subject variability is zero, which requires the unrealistic scenario of an infinite number of trials. Given these results, the conclusions in Grange and Schuch (2023) appear to be based on a simulation setup that neither incorporated between-subject variability nor examined the interplay between between- and within-subject correlations – both of which help explain the outcomes of their Simulations 2–5.

**Fig. 4:**
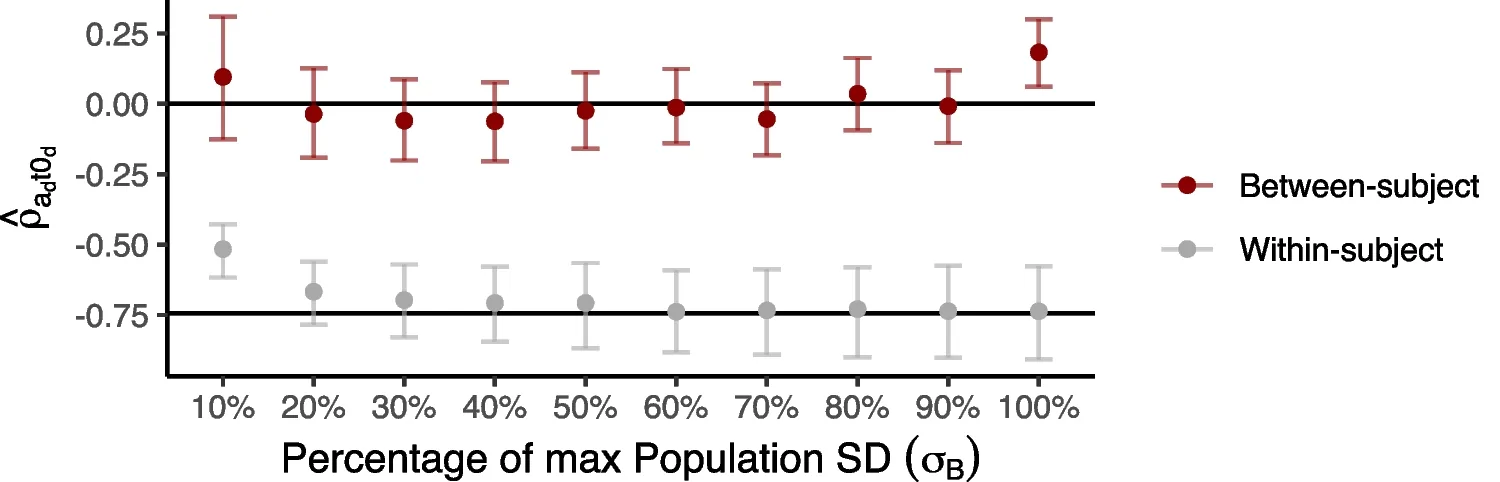
Posterior medians (*red bullets*) and 95% credible intervals (*red whiskers*) of the between-subject correlations of the participant-level $t0_{d}$ and $a_{d}$ parameters for varying extents of between-subject variability, as estimated by a hierarchical DDM. The within-subject correlations are shown in *gray*, and are estimated from the correlations in the participant-level joint posterior distribution of the difference scores; the *bullets* show the median across participants, with the *error bars* representing the 2.5%- and 97.5% quantiles. The horizontal lines represent the true between-subject correlation of zero and the spurious correlation of -.743 reported by Grange and Schuch (2023) in Simulation 1. At 100%, the data were simulated with the upper bound of the population standard deviation of the difference scores reported in Table 1, and at 10%, they were simulated with a tenth of that upper bound

In Simulation 2, Grange and Schuch (2023) reported that the strongly negative spurious correlation between $a_{d}$ and $t0_{d}$ emerged only when *v* was manipulated across conditions. In this setup, *v* was sampled from a normal distribution while $a_{d}$ and $t0_{d}$ were again fixed at zero for all participants, thereby removing all between-subject variability. As a result, the same issues outlined above apply. In contrast, manipulating *a* or *t*0 – that is, sampling their values and thus inducing individual differences in at least one difference score – reduced the observed correlation between $a_{d}$ and $t0_{d}$. This occurred because the between-subject correlation carried more weight, as a larger proportion of the total variance was attributable to between-subject variability (see Equations 1-3). In line with this explanation, when Grange and Schuch (2023) sampled all three parameters from normal distributions, the spurious correlation between the difference scores was not found. Simulations 3-5 all build on the data-generation setup from Simulation 1 and therefore produce essentially the same results, regardless of the type of software, the kind of evidence-accumulation model, or the value of the true data-generating between-subject correlation.

It is worth noting that a simulation setup, which omits between-subject variability in the difference scores, may be somewhat unrealistic for empirical data. By Grange and Schuch (2023)’s own recommendations, the appropriate analytic approach would involve model selection. In the simulations without condition differences, this would lead to constrained parameters across conditions, so difference scores would not even be computed. In the simulations with condition differences, this would reveal that parameters need not vary across individuals, in which case correlating difference scores would be unnecessary. Nevertheless, our extension of the authors’ simulations raises an important question, that is: how can researchers determine whether the between-subject variability in model parameters is sufficient to minimize the effect of within-subject correlations on the observed correction? Although Monte Carlo simulations like those used by Ratcliff and Tuerlinckx (2002) can help gauge the relative importance of between- and within-subject correlations, they can never provide a definitive answer – especially not a priori, before data have been collected and fit with the cognitive model in question. Fortunately, as we will show, an appropriately parameterized hierarchical model can safeguard against drawing erroneous conclusions in the presence of within-subject correlations, even when the between-subject variability of the model parameters is negligible.

### Hierarchical estimation safeguards against spurious correlations

Hierarchical modeling (Gelman & Hill, 2007) simultaneously estimates cognitive model parameters at the participant level and their distribution at the population level, thereby accounting for both the within-subject and between-subject variance of the parameters. In doing so, it can effectively disentangle within- and between-subject correlations in cognitive models with parameter trade-offs.

#### Simulations

We fit a subset of the previously simulated datasets with a Bayesian hierarchical implementation of the simple three-parameter DDM (Stone, 1960) using the R package *EMC2* (Stevenson et al., 2026). We focused on the dataset with 1000 trials per condition and without population-level effects. We did not fit the dataset with zero between-subject variability in the difference scores since this would result in a misspecified model and would lead to convergence problems. To reduce computational burden, we used the first 250 of the 1000 generated participants, as this is more than sufficient to inform the population-level distributions and the corresponding population-level parameters.

We parametrized the model in terms of differences between the *Hard* and the *Easy* conditions, e.g., $t0_{hard} = t0 + 0.5 \times t0_{d}$ and $t0_{easy} = t0 - 0.5 \times t0_d$. Importantly, we explicitly modeled all participant-level parameters, including $a_{d}$, $t0_{d}$, and $v_{d}$, as coming from a multivariate normal population-level distribution, which allows for the estimation of the variance-covariance matrix of all parameters. See the Appendix A for the complete hierarchical model specification, including the priors. In doing so, we avoided the two-step procedure of first estimating the cognitive model parameters and then computing the difference scores and their correlations outside the model. Instead, we draw inferences directly from the posterior distribution of the between-subject correlation of the difference scores, uncontaminated by within-subject correlations. We used *EMC2*’s default prior setting for the hyperparameters (i.e., standard normals on the transformed scale). The variance-covariance matrix of the participant-level parameters was assigned a non-informative prior that implied a uniform distribution (-1,1) on the correlations (Huang & Wand, 2013). We used *EMC2*’s default fitting routine, ensuring that the Gelman–Rubin potential scale reduction factor was below 1.1 (Stevenson et al., 2026). We ran four chains and ended up using 4000 MCMC samples for inference (1000 per chain).

#### Results

The red symbols in Fig. 4 show the medians and 95% credible intervals of the posterior distributions of the estimated correlations between $t0_{d}$ and $a_{d}$ for varying extents of between-subject variability in the difference scores. For each participant in each dataset, we also computed the within-subject correlation between $t0_{d}$ and $a_{d}$ using the joint posterior distribution of the participant-level parameters – the gray symbols show the medians and the 2.5% and 97.5% quantiles across participants for each dataset. As opposed to the two-step approach, the hierarchical analysis correctly located the between-subject correlations to be around zero (red bullets). The spurious correlation of approximately -.70 reported by Grange and Schuch (2023) is confined to the within-subject correlations (gray bullets).

**Fig. 5:**
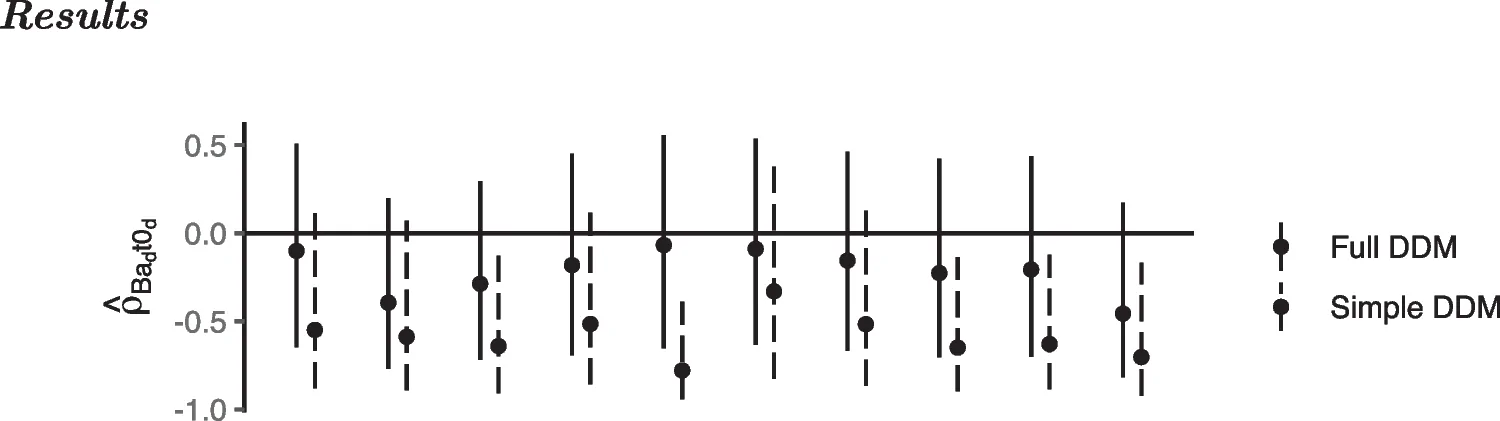
Posterior medians and 95% credible intervals of the between-subject correlations of the participant-level $t0_{d}$ and $a_{d}$ parameters across ten repetitions of Dutilh et al. (2019)’s Experiment 1. The datasets were fit using a hierarchical implementation of the full (*solid lines*) and simple three-parameter DDM (*dotted line*). Given the random assignment of trials to conditions, the true between-subject correlation between the difference scores should be zero

#### Discussion

Using subsets of the simulated datasets from the previously reported EZ analysis, we showed that a hierarchical DDM that explicitly models the variance-covariance matrix of the estimated parameters can successfully disentangle between-subject and within-subject correlations, regardless of the degree of between-subject variability in the model parameters – or in this case the differences between model parameters. Importantly, achieving this requires explicitly accounting for the variance-covariance matrix of the individual-level parameters within a single hierarchical model.

The hierarchical analyses reported by Grange and Schuch (2023) highlight the key role of the latter detail. In one of their supplementary analyses, they employed hierarchical modeling but still found spurious correlations. However, a hierarchical DDM was applied to the *Easy* and *Hard* conditions separately, estimating two independent models, without accounting for the covariance of the model parameters within and between conditions – just as in the EZ analyses. The posterior means of the participant-level estimates were then extracted, the difference scores were computed, and these scores were correlated. This two-step approach is unable to decompose the total (co)-variance into between- and within-subject components, underscoring the need to explicitly account for the covariance structure of the participant-level parameters within the same hierarchical model.

Note that the hierarchical DDM used here is severely overparametrized, since there were no differences in parameters across conditions. In real applications, one would first test whether allowing parameters to vary across conditions is necessary at all, using model selection criteria such as Bayes factors (Gronau, Heathcote, & Matzke, 2020; Kass & Raftery, 1995), the Deviance Information Criterion (Spiegelhalter, Best, Carlin, & van der Linde, 2002), or similar metrics. In this case, such an approach would likely have favored a simpler model with parameters constrained to be equal across conditions. Consequently, no difference scores would have been estimated in the first place, avoiding inference about the correlation between difference scores altogether.

### Empirical example

Grange and Schuch (2023) identified a spurious negative correlation between $t0_{d}$ and $a_{d}$ not only in simulated data but also in Experiment 1 of the many-analysts project by Dutilh et al. (2019), which included 20 participants and 468 trials of a random dot motion task in a single condition. Grange and Schuch (2023) randomly assigned each trial within participants to one of two artificial conditions, A and B, then fit a simple three-parameter DDM (Stone, 1960) to each condition and each participant separately using maximum likelihood estimation (*fast-dm-30;* Voss et al., 2015). They then calculated, for each participant, A–B difference scores between the model parameters, and correlated them with each other. Given the random assignment of trials to conditions, no systematic between-subject relationship between the difference scores should exist, meaning any observed correlation would be purely spurious. Nevertheless, they still found a strong negative correlation of r=-.964 using this two-step approach.

We repeated the random assignment procedure used by Grange and Schuch (2023) ten times with the same random seed as the authors. The only modification that we made to the data pre-processing was the exclusion of very fast, likely anticipatory, responses prior to modeling, specifically, RTs equal to or faster than 0.2 s (22 observations; 0.24% of the data) as those can bias parameter estimates (Ratcliff & Tuerlinckx, 2002)^7^. We then fit two versions of the hierarchical DDM to the ten datasets, again using the same difference parameterization as in our simulations (e.g., $t0_{hard} = t0 + 0.5 \times t0_{d}$ and $t0_{easy} = t0 - 0.5 \times t0_d$; see the Appendix A for the full model specification, including priors). The first version, the “full” DDM (Ratcliff & McKoon, 2008) , estimated not only *v*, *a*, *t*0, and corresponding difference scores, but also relative start point bias *z*, relative between-trial variation in start point *sz*, between-trial variation in the drift rate *sv*, and between-trial variation in non-decision time *st*0, including the corresponding difference scores. The second version was the hierarchical implementation of the simple three-parameter DDM with only *v*, *a*, *t*0, and their difference scores, as used in our simulations. This is the version used by Grange and Schuch (2023) to model the Dutilh et al. (2019) data, albeit in a non-hierarchical fashion. Given the smaller trial size, we used slightly more informative priors on the population-level means. The implied prior on the correlation was again uniform from -1 to 1 (Huang & Wand, 2013).

#### Results

**Fig. 6:**
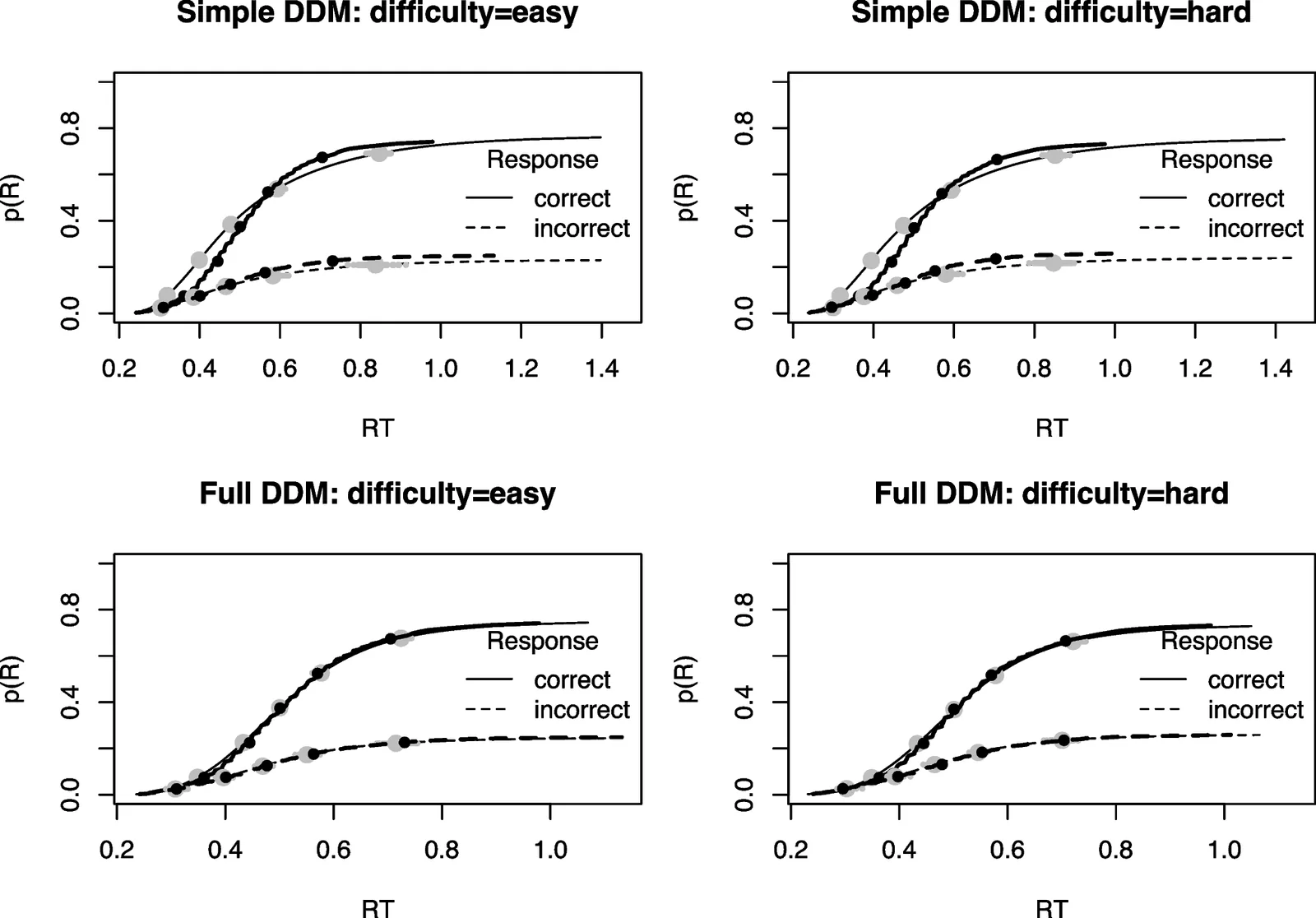
Posterior predictive assessment of the simple three-parameter and full seven-parameter hierarchical DDM, corresponding to the fifth repetition of Dutilh et al. (2019)’s Experiment 1. The plots show predicted (*gray*; including 95% credible intervals) and observed (*black*) defective cumulative distribution functions averaged across participants for correct (*solid*) and incorrect (*dashed*) responses. The predictions are based on 100 randomly selected posterior samples from the joint posterior distribution of the model parameters. The *dots* represent the $10^{th}$, $30^{th}$, $50^{th}$, $70^{th}$, and $90^{th}$ percentiles of the response time (RT) distributions. p(R) = probability of a response

Figure 5 shows that for the full DDM, the 95% credible intervals of the correlation between $t0_d$ and $a_d$ consistently include zero across repetitions. However, in the three-parameter DDM, there is a clear downward bias, though its extent varies across repetitions. Figure 6 illustrates the likely cause of this pattern of results: a failure of the simple DDM to adequately describe the observed data. The figure compares the observed data with predictions generated from the joint posterior distribution of the parameters of the two models (i.e., posterior predictive assessment). In contrast to the full DDM, the simple DDM is unable to account for the observed data, drastically overestimating slow RTs for incorrect and correct responses and underestimating fast RTs for correct responses in both conditions.

#### Discussion

While the simple DDM analysis using the two-step approach reported in Grange and Schuch (2023) of Dutilh et al. (2019)’s Experiment 1 found a strong spurious negative correlation, the full hierarchical DDM correctly estimated the between-subject correlation between $a_{d}$ and $t0_{d}$ to be around zero. Notably, the poorly fitting hierarchical three-parameter DDM still led to a downward bias in the correlation, underscoring the importance of carefully evaluating a model’s descriptive accuracy before using it for statistical inference. Note again that both the simple and the full DDM used here are severely overparameterized given the absence of condition differences; they serve merely illustrative purposes. In real applications, model selection – along with theoretical considerations – should guide researchers in selecting the model that strikes the best balance between parsimony and descriptive accuracy.

## General discussion

In this paper, we demonstrated how and when within-subject correlations between parameters of cognitive models can affect correlations computed between parameter estimates across participants. Through simulations using the EZ diffusion decision model (DDM), we demonstrated that observed correlations between parameter estimates are a weighted combination of the within-subject and true between-subject correlations of the parameters. In particular, we showed how within-subject correlations can dominate the observed correlation when the two-step approach is used, where the cognitive model is first fit to participants’ data, and correlations are then computed on the resulting point estimates. The influence of the within-subject correlation on the observed correlation is determined by the degree of between-subject variability in the parameter estimates and the number of trials per participant. We then demonstrated that simultaneously modeling the participant-level data and the variance-covariance structure of the participant-level cognitive model parameters using hierarchical (multilevel) modeling can effectively disentangle between- and within-subject correlations (see also Hamaker, Asparouhov, Brose, Schmiedek, & Muthén, 2018; Haslbeck & Epskamp, 2023; McNeish & Hamaker, 2020). Lastly, through a re-analysis of empirical data, we demonstrated that the descriptive accuracy of the cognitive model plays a crucial role in successfully teasing apart the (co)variances of model parameters. Note that although our case study – following Grange and Schuch (2023) – focused on correlations involving differences between model parameters, the same conclusions apply to correlations between the parameters themselves.

Our results show that, for cognitive models that exhibit strong within-subject parameter trade-offs, two-step procedures that do not simultaneously model the participant-level data and the variance-covariance structure of the parameters should not be used in applications that involve inference about correlations. Although rules of thumb exist regarding the relative size of between-and within-subject variability needed to safeguard inference (e.g., Ratcliff & Tuerlinckx, 2002), we argue that the most sensible approach is to avoid two-step procedures altogether and estimate an appropriately parametrized hierarchical model that includes the variance-covariance matrix of all parameters of interest.

Until recently this recommendation was impractical for evidence-accumulation models in general, and the full DDM in particular, because evaluating the likelihood function is computationally costly and parameter trade-offs complicate Bayesian sampling. As we demonstrated, these issues are addressed by recent advances in Bayesian sampling methods (Gunawan, Hawkins, Tran, Kohn, & Brown, 2020; Hartmann & Klauer, 2021) implemented in the *EMC2* package (Stevenson et al., 2026), which also makes it easy to flexibly specify the required parameterizations and obtain parameter estimates and model selection metrics within practical computation times. (see Stevenson et al., 2026, for speed comparisons between different software). Bayesian hierarchical modeling of correlations in simpler cognitive models can be implemented relatively easily in standard MCMC software (e.g., Matzke, Dolan, Batchelder, & Wagenmakers, 2015; Stan Development Team, 2025).

The use of hierarchical modeling to separate within- and between-subject correlations has been also recommended in the time-series literature (e.g., Asparouhov, Hamaker, & Muthén, 2018; Epskamp, 2020; Hamaker et al., 2018; Haslbeck & Epskamp, 2023; McNeish & Hamaker, 2020). Approaches such as multilevel vector autoregression (VAR) models or dynamic structural equation models jointly estimate within- and between- participant variability, thereby reducing the risk of spurious conclusions.

When the primary objective is to maximize power to detect differences between groups (van Ravenzwaaij, Donkin, & Vandekerckhove, 2017), it has been recommended to use the simpler EZ model rather than the full DDM – potentially at the cost of descriptive accuracy. However, a note of caution is warranted for individual-differences research. Our results underscore that ensuring adequate model fit is essential when drawing inferences from correlations between parameter estimates, also when using appropriately parameterized hierarchical models. Future research should examine in more detail which aspects of model misfit drive this effect and how they interact with estimation bias, although such investigations are likely to be both model- and design-specific.

Although the current paper focused on evidence-accumulation models, parameter trade-offs do not only occur in cognitive models, but also in many simpler statistical models. Sloppiness can arise in any multi-parameter model where parameters are estimated with high uncertainty and can vary across a relatively wide range without significantly affecting model fit (Gutenkunst et al., 2007; Waterfall et al., 2006). Widely used examples of models with pronounced parameter trade-offs include the ex-Gaussian distribution (Matzke & Wagenmakers, 2009; Schmiedek et al., 2007), simple regression (e.g., Ratcliff & Tuerlinckx, 2002), and polynomial regression (Waterfall et al., 2006).

## Conclusion and recommendations

Strong within-subject correlations between model parameters are a common characteristic of cognitive models. These within-subject correlations can contaminate correlations between parameter estimates obtained via non-hierarchical methods. We have shown that an appropriately parameterized and descriptively adequate hierarchical model that explicitly includes the variance-covariance matrix of the cognitive model parameters efficiently disentangles within-and between-subject correlations and protects against spurious correlations. We recommend the following workflow: (1) whenever possible, specify theoretically or empirically informed prior distributions for the model parameters (2) estimate the model hierarchically including the variance-covariance structure of the model parameters, (3) evaluate the convergence of the sampling algorithm and the descriptive accuracy of the model, (4) conduct model selection to remove unnecessary complexity, and (5) perform prior sensitivity analyses when prior choices are arbitrary. Although frequentist hierarchical methods can, in principle, be used for this purpose, in practice, the Bayesian framework is often the only computationally feasible option. When hierarchical modeling is infeasible, we recommend using a large number of trials per participant and condition, which reduces within-subject variability and, consequently, the extent to which the within-subject correlation contributes to the observed correlation between the estimates.

## Data Availability

The analyzed archival empirical data (Dutilh et al., 2019) are available at https://osf.io/z5nqm.

## Appendix A

### Model specification: Hierarchical simulation

Let $Y_{ilk}$ denote participant i’s response vector (reaction time and accuracy) in condition $x_l = -0.5, 0.5$ for trial k:

4 $$\begin{aligned} Y_{ilk} \sim {\text {DDM}}\!\bigl (t_{0,il}, a_{il}, v_{il}\bigr ). \end{aligned}$$

*EMC2* estimates the non-decision time parameters t0 and boundary separation parameters a on the log scale, as they are constrained to be positive (Stevenson et al., 2026). The drift rate v is estimated on the real line, as it can take negative values:

$$ \begin{aligned} t_{0,il}&= \exp \!\bigl (t_{0,i} + x_{l}\, t_{0d,i}\bigr ), \\ a_{il}&= \exp \!\bigl (a_{i} + x_{l}\, a_{d,i}\bigr ), \\ v_{il}&= v_{i} + x_{l}\, v_{d,i}. \end{aligned} $$

Let $\theta _i = [t_{0,i}, t_{0d,i}, a_i, a_{d,i}, v_i, v_{d,i}]^\top$ denote the vector of participant-specific parameters. We assume these follow a multivariate normal distribution with population-level mean vector $\boldsymbol{\mu }$ and variance–covariance matrix $\boldsymbol{\Sigma }$:

$$ \theta _i \sim \mathcal {MVN}(\boldsymbol{\mu }, \boldsymbol{\Sigma }). $$

The population-level parameters are defined as

$$\begin{aligned} \boldsymbol{\mu }= &  \begin{bmatrix} \mu _{t_0} \\ \mu _{t_{0d}} \\ \mu _a \\ \mu _{a_d} \\ \mu _v \\ \mu _{v_d} \end{bmatrix},\\ \boldsymbol{\Sigma }= &  \begin{bmatrix} \sigma ^2_{Bt_0} & \sigma _{Bt_0,t_{0d}} & \sigma _{Bt_0,a} & \sigma _{Bt_0,a_d} & \sigma _{Bt_0,v} & \sigma _{Bt_0,v_d} \\ \sigma _{Bt_0,t_{0d}} & \sigma ^2_{Bt_{0d}} & \sigma _{Bt_{0d},a} & \sigma _{Bt_{0d},a_d}& \sigma _{Bt_{0d},v} & \sigma _{Bt_{0d},v_d} \\ \sigma _{Bt_0,a} & \sigma _{Bt_{0d},a} & \sigma ^2_{Ba} & \sigma _{Ba,a_d} & \sigma _{Ba,v} & \sigma _{Ba,v_d} \\ \sigma _{Bt_0,a_d} & \sigma _{Bt_{0d},a_d} & \sigma _{Ba,a_d} & \sigma ^2_{Ba_d} & \sigma _{Ba_d,v} & \sigma _{Ba_d,v_d} \\ \sigma _{Bt_0,v} & \sigma _{Bt_{0d},v} & \sigma _{Ba,v} & \sigma _{Ba_d,v} & \sigma ^2_{Bv} & \sigma _{Bv,v_d} \\ \sigma _{Bt_0,v_d} & \sigma _{Bt_{0d},v_d} & \sigma _{Ba,v_d} & \sigma _{Ba_d,v_d} & \sigma _{Bv,v_d} & \sigma ^2_{Bv_d} \end{bmatrix}. \end{aligned}$$

Given the large number of trials per condition and participant (K=1000), weakly informative hyperpriors were used. Following *EMC2*’s defaults, the population-level means $\boldsymbol{\mu }$ were assigned standard normal priors:

$$ \boldsymbol{\mu } \sim \mathcal {N}(0,1). $$

For the variance–covariance matrix $\boldsymbol{\Sigma }$, *EMC2*’s default scale-mixture inverse-Wishart prior was used, with mixture weights for each parameter following inverse-Gamma distributions (Huang & Wand, 2013; Stevenson et al., 2026):

$$ \begin{aligned} \boldsymbol{\Sigma } \mid a_{t_0}, \ldots , a_{v_d}&\sim \text {Inverse-Wishart}\\&\left( v + p - 1,\; 2v\,\text {diag}(1/a_{t0}, \ldots , 1/a_{v_d})\right) , \\ a_{t_0}, \ldots , a_{v_d}&\sim \text {Inverse-Gamma}\!\left( \tfrac{1}{2}, 1/A^2\right) , \end{aligned} $$

where p=6, v=2, and A=0.3, which yield approximately uniform marginal correlations. *p* reflects the dimensionality of the variance-covariance matrix, and the prior on $\boldsymbol{\Sigma }$ implies Half-t(*v*, *A*) distributions for the between-subject variability parameters $\sigma _{Bt_0}, \ldots , \sigma _{Bv_d}$.

### Model specification: Empirical example

In the empirical example, the specification of the simple, three-parameter DDM was identical to the hierarchical model described above, except that the hyperpriors on the population-level means were specified to be more informative:

$$ \begin{aligned} \mu _{t_0} \sim \mathcal {N}(-0.9, 0.6) \\ \mu _{t_{0d}} \sim \mathcal {N}(0, 1) \\ \mu _a \sim \mathcal {N}(1, 0.6) \\ \mu _{a_d} \sim \mathcal {N}(0, 1) \\ \mu _v \sim \mathcal {N}(2, 2) \\ \mu _{v_d} \sim \mathcal {N}(0, 1). \end{aligned} $$

The specification of the full DDM was again identical to the hierarchical model described above, but also included the relative start-point bias *z* and the between-trial variability parameters. Note that *z* and relative between-trial variation in start point *sz* are estimated on the probit scale, and between-trial variation in drift rate *sv* and non-decision time *st*0 on the log scale. The hyper priors for the additional population-level means were:

$$ \begin{aligned} \mu _{sv} \sim \mathcal {N}(0, 1) \\ \mu _{sv_{d}} \sim \mathcal {N}(0, 1) \\ \mu _{sz} \sim \mathcal {N}(0, 0.7) \\ \mu _{sz_d} \sim \mathcal {N}(0, 0.5) \\ \mu _{st0} \sim \mathcal {N}(-3, 1.1) \\ \mu _{st0_d} \sim \mathcal {N}(0, 1) \\ \mu _{z} \sim \mathcal {N}(0, 0.2) \\ \mu _{z_d} \sim \mathcal {N}(0, 0.4) \\ \end{aligned} $$

For both the full and the simple DDM, *EMC2*’s default prior with implied uniform between-subject correlations was used for the variance-covariance matrix. For the full DDM, p=14 since 14 parameters were estimated for each participant.

## Footnotes

1 Yet another approach to avoid spurious conclusions is to eliminate or reduce within-subject dependencies by re-parameterizing the cognitive model (e.g., Krefeld-Schwalb et al., 2022; Scheibehenne & Pachur, 2015). However, such dependencies are often inherent to cognitive models, and even where they can be reduced, doing so likely conflicts with estimating psychologically meaningful parameters, which is undesirable in terms of interpretability.

2 Starting point *z* was set to 0.5*a*, corresponding to unbiased responding.

3 Grange and Schuch (2023)’s Simulation 5 used the same parameterization

4 A few parameter sets had to be regenerated because some parameters were outside the theoretically possible range (e.g., negative boundary separation *a*).

5 The exception was $v_{easy}$ and $v_{hard}$, which we did not constrain to be positive

6 We thank the authors for providing well-documented code.

7 To confirm that the strong spurious correlation was not driven by contaminant RTs, we conducted EZ analyses using the two-step approach both with and without excluding these observations. The observed correlation between the difference scores was virtually unchanged: r=-.921, 95% CI [-.969, -.808] in the cleaned data and r=-.925, 95% CI [-.970, -.817] without exclusion.

## Code availability

All simulation and analysis code as well as the MCMC samples are available at https://osf.io/z5nqm.
